# Outcomes of Hip Arthroscopy in Patients with Femoroacetabular Impingement and Concomitant Tönnis Grade II Osteoarthritis or Greater: Protocol for a Systematic Review

**DOI:** 10.29337/ijsp.26

**Published:** 2021-03-16

**Authors:** Octavian Andronic, Leica Claydon, Rachael Cubberley, Karadi Hari Sunil-Kumar, Vikas Khanduja

**Affiliations:** 1 Department of Orthopaedics, Balgrist University Hospital, University of Zürich, Zürich, Switzerland; 2 Faculty of Health, Education, Medicine, and Social Care, Anglia Ruskin University, United Kingdom; 3 Young Adult Hip Service, Department of Trauma and Orthopaedics, Addenbrooke’s Hospital, Cambridge, United Kingdom

**Keywords:** hip arthroscopy, femoroacetabular impingement, FAI, hip osteoarthritis, hip preservation, outcomes

## Abstract

**Introduction:**

Outcomes of hip arthroscopy for femoroacetabular impingement and concomitant moderate- to advanced hip osteoarthritis (Tönnis Grade II or greater) is still a matter of debate as findings in the literature are controversial. This study aims to investigate whether hip arthroscopy is effective in treating patients with femoroacetabular impingement and Tönnis hip osteoarthritis Grade II or greater.

**Methods and Analysis:**

The protocol follows the PRISMA-P guidelines. The systematic review is registered in the International Prospective Register for Systematic Reviews and Meta-analysis (PROSPERO) under the registration number: CRD42020210936. The search will include multiple databases: MEDLINE, EMBASE, Web of Science Core Collection and Cochrane library. The screening and selection process will be performed by two independent researchers based on predefined criteria. All studies published in English or German from inception to 1^st^ of December 2020 that investigated outcomes of hip arthroscopy in patients with Tönnis grade II or greater of hip osteoarthritis will be considered eligible. The risk of bias and quality of articles will be assessed using the MINORS tool. Methodological inconsistency and heterogeneity will be explored using the I^2^ test. This assessment will be used to provide recommendations using the GRADE system.

**Ethics and Dissemination:**

Separate ethical approval is not required. This study will be a comprehensive and rigorous systematic review on all published articles reporting on outcomes of hip arthroscopy for femoroacetabular impingement and concomitant hip osteoarthritis Tönnis Grade II or greater. It will explore patient reported outcomes as well as radiological outcomes, complications, rates of revision surgery and rates of conversion to total hip replacement (THR). Results of the current review will be published in a peer-reviewed scientific journal and disseminated on research platforms according to copyright rules and rights.

**Highlights:**

- Hip arthroscopy is used to treat femoroacetabular impingement and is effective in patients that have concomitant hip osteoarthritis Tönnis Grade 0 or 1.
- Outcomes of hip arthroscopy in patients with femoroacetabular impingement and in moderate to advanced osteoarthritis – Tönnis Grade 2 or greater, is a matter of debate.
- The purpose of the current systematic review is to elucidate, stratify and critical appraise the current evidence on outcomes in this patient subpopulation.

## 1. Introduction

Femoroacetabular impingement (FAI) is a condition characterized by an abnormal hip morphology and represents an important cause of hip pain in young adults with an increasing interest worldwide [1,2]. It is hypothesized that femoroacetabular impingement may lead to earlier development of hip osteoarthritis (OA) [3]. FAI is treated either conservatively with activity-modification and physiotherapy [4] or with joint-preservation surgery, mainly hip arthroscopy (HA) or in specific cases, periacetabular osteotomies (PAO) or surgical hip dislocations [5]. The aim of such surgery is to reshape the hip joint to prevent impingement (abnormal mechanical contact) [6] and normalise stresses in the hip joint [7] and also address any concomitant extra-articular causes if present [8,9]. Intra-articular damage is addressed with debridement, repair or reconstruction in cases of labral tears, and a debridement or microfracture with different biological regenerative techniques in cases of articular cartilage damage [10]. It is now been well-reported, that HA is effective in achieving clinical improvement in patients with FAI and concomitant OA of Tönnis Grade I, demonstrated by the results of large scale multicentre randomized controlled trials (RCT): the UK FASHIoN (RCT) [11] and the FAIT trial [12].

However, outcomes for FAI and concomitant moderate- to advanced hip osteoarthritis (Tönnis Grade II or greater), for both hip arthroscopy or conservative regimens, is still a matter of intense debate as the best treatment remains controversial [13,14]. As such, it is still unclear whether patients with FAI and signs of osteoarthritis would benefit from preservation surgery or should with conservative management or a joint replacement [5]. An important concern in young adults receiving a total hip replacement (THR), is the probability that they will most likely require multiple revisions in their lifetime [15], leaving the ultimate long-term outcome questionable. It is also unclear, what amount of OA can be managed with hip preservation techniques and which kind of surgery with what types of augmentation, should be employed.

This study, therefore, aims to investigate whether hip arthroscopy is effective in treating patients with femoroacetabular impingement and concomitant Tönnis hip grade II OA or greater.

## 2. Methods and Analysis

### 2.1 Study design

The systematic review will follow The Preferred Reporting Items for Systematic Reviews and Meta-analysis – Protocol (PRISMA-P) guidelines [16]. The PRISMA statement will be used for the development of a flowchart of the systematic search. This systematic review is registered in the International Prospective Register for Systematic Reviews and Meta-analysis (PROSPERO) under the registration number: CRD42020210936.

### 2.2 Search strategy

Several databases will be searched, including: MEDLINE, EMBASE, Web of Science Core Collection and the Cochrane library. It is expected that about 50% of studies to be extracted from MEDLINE, whilst Web of Science Core Collection will cover the grey literature. Moreover, the reference lists will be searched at the full-text assessment stage. Articles published in English or German language from database inception to December 1^st^ 2020 will be included. The search key-words will include studies looking at patients that underwent hip arthroscopy for the treatment of femoroacetabular impingement and concomitant hip osteoarthritis Tönnis Grade II or greater and will then be combined using the Boolean terms AND/OR. Combinations for each database using the following key-words will be used: “hip arthroscopy”, “FAI”, “femoroacetabular impingement” and “outcome”, “failure”, “results”.

### 2.3 Study selection

A blinded and independent process of study selection based on title and abstract will be performed by two authors (OA and SK). Next, further selection based on predefined criteria will be performed by evaluating full texts. Studies will be screened according to the inclusion and exclusion criteria (***Table 1***). Review articles, surgical techniques, oral presentations, experimental or animal studies, studies mixing and overlapping patient populations will be excluded. The PICO tool has been used to formulate the research question. The participants will be young adults older than 16 years of age and younger than 45 years of age. This will allow exclusion of the paediatric population where the growth plates may still be open [17] and where joint degeneration would be less likely to occur without secondary causes [18] and also of older patients, where it is well-known, that age alone, is a negative predictive factor of the outcome of hip arthroscopy [19,20]. Participants with active inflammatory disease, neurological conditions, previous ipsilateral surgeries of the hip or osteonecrosis will be excluded.

**Table 1:** Inclusion and Exclusion Criteria based on PICO tool.


INCLUSION CRITERIA	EXCLUSION CRITERIA

Study Design: Original articles in English or German language Population: Patients with femoroacetabular impingement and Tönnis grade of hip osteoarthritis II or greater. Patients older than 16 years of age and younger than 45 years of age. Sample size equal or greater to 10 hips Intervention: Hip arthroscopy alone as the only intervention without open surgery Comparators: Patients with FAI with either Tönnis ≤I that received hip arthroscopy or with Tönnis ≥II that received any type of conservative treatment Outcomes: Patient reported outcomes, rates of conversion to total hip replacement (THR), complications, radiological outcomes. Minimum follow-up of 6 months	Study Design: Review articles, surgical techniques, oral presentations, experimental or animal studies. Articles not written in English or German Population: Patients with osteonecrosis or post-avascular necrosis sequelae (Perthes), slipped capital femoral epiphysis, inflammatory or septic arthritis, previous surgery on the ipsilateral hip Intervention: Intervention included adjunct open procedure


### 2.4 Data extraction

The selected articles will then be exported to the Mendeley reference manager software and all duplicates will be removed. The final number of included articles will then be assessed for full text review and data will be extracted based on a pre-determined set of variables. Two reviewers (AO and SK) will assess and screen and if there is any discrepancy, a third more senior reviewer (VK) will be invited to advice until consensus is achieved between all authors. Data will be extracted into Microsoft Excel v16.21 under the following headings: author, study setting (country and year), number of included hips, follow-up, gender, age, Body Mass Index (BMI), diagnosis, stage of disease, previous treatments, surgical technique, augmentation technique (if any), clinical outcome (with preoperative and postoperative results where applicable), radiographic outcome, complications, revision rates, rates of conversion and time to joint resurfacing or total joint replacement (THR).

### 2.5 Data analysis and synthesis

The risk of bias and quality of studies will be evaluated using the MINORS criteria [21] (Methodological index for non-randomized studies) for each study design due to its rigor in assessing the methodological integrity of studies and also due to the retrospective nature of studies that exist in the literature. The process will be performed independently by two reviewers (AO and SK) and the senior author will be consulted in case no consensus is reached (VK). MINORS criteria assess eight critical aspects of study design for non-comparative clinical studies and an additional four aspects of study design for comparative clinical studies. Each item is given a score of zero if information is not reported, one if information is reported but inadequate, and two if information is reported and adequate. Therefore, the maximum possible score is 16 for comparative studies and 24 for non-comparative studies. Each score was then converted into a percentage to harmonise the scoring system.

Based on the scoring on quality assessment, the cumulative confidence in evidence from all the data collected will be assessed using the Grading of Recommendations Assessment, Development and Evaluation (GRADE) system to inform recommendations [22].

Furthermore, an additional quality assessment for studies reporting on biological augmentation, mainly with MSCs (mesenchymal stem cells) will be performed using the MIBO tool [23] (Minimum Information for Studies Evaluating Biologics in Orthopaedics). A scoring system was then used per study such as studies that answered yes to a question from the checklist scored 2, not clear scored 1 and no scored 0. Each score was then converted into a percentage to harmonise the scoring system.

The statistical analysis will be performed using Review Manager (RevMan Cochrane) and Comprehensive Meta-Analysis Software (CMA). Extraction and comparison will be performed by two authors independently (OA, SK). Any discrepancy will be consulted with the last author (VK) until an agreement will be reached.

For further quantification of methodological inconsistency and heterogeneity across studies, a I² test will be performed, with a p-value of p = 0.10. Values more than 40% are considered significant for moderate heterogeneity and over 75% are considered to be highly heterogeneous. This will assess whether observed differences in results are compatible with chance alone.

Depending on the heterogeneity, a random-effects-model will be used for the meta-analysis if I² will be not significant, p > 0.1, otherwise, a fixed-effect model will be employed. Meta-analysis will be performed if the level of evidence of included studies will allow such analysis. If possible, outcomes will be longitudinally assessed at different timepoints of follow-up postoperatively: 6 months, 12 months and 24 months.

Two subgroup analysis will be performed based on the scores achieved on quality assessment and based on disease stage, respectively. Studies will be separately analysed in two groups: those that achieved more than 50% using MINORS and those that achieved 50% or less. The second subgroup analysis will stratify the outcomes based on preoperative disease stages: FAI with Tönnis ≤I versus FAI with Tönnis ≥II.

## 3. Discussion

Femoroacetabular impingement (FAI) has a significant negative economic impact [24]. Patients affected by the pathological process are usually young and are in the most economically productive period of their life. Therefore, being debilitated by the condition and being treated conservatively or with surgical management after unsuccessful non-operative treatment has a great impact on productivity [25,26].

Hip arthroscopy has been shown to reduce the substantial economic burden on patients suffering from FAI through indirect cost savings in a patient cohort in the United States of America [24]. Reports have shown that a rehabilitation of 3 months to 1 year is required to achieve an improvement compared to the baseline pre-operative state, with however, some remaining deficits in activity [27,28].

However, the efficacy of hip arthroscopy as a treatment option for patients with femoroacetabular impingement and concomitant hip OA remains a challenging subject without clear answers. The amount of degenerative changes that are already present and would preclude any benefits from surgery, are still to be elucidated. The purpose of this study is to determine whether moderate to advanced pre-operative concomitant OA precludes benefit of hip arthroscopy by systematically reviewing the literature on outcomes of hip arthroscopy for FAI in the setting of OA.

It is already known that there is a beneficial role of hip arthroscopy in patients with mild OA in terms of patient reported outcomes, as reported by two large scale multicentre randomized controlled trials [11,12]. Furthermore, contraindications for hip arthroscopy due to poor outcomes have been previously described: less than 2 mm of joint space and Tönnis grade III changes [14,29]. However, this leaves the patient cohort with FAI and Tönnis Grade II OA or greater, and literature is filled with contradictory outcomes and the debate on the best way of managing this cohort of patients continues [13,30]. Domb and colleagues [30] have systematically reviewed the data and reported a 23% conversion rate to THR among patients with hip OA compared with 8.3% among patients without OA at a mean follow-up of 32 months (range, 24 to 54 months). Supporting these findings, the same authors reported a 41% conversion rate to THR in their patient cohort with grade II Tönnis changes compared with a 11% conversion rate to THR at 2 years among patients with grade 0 and I Tönnis OA [31]. Opposed to these results, Byrd et al. stated no differences in terms of conversion to THR between patients with FAI and Tönnis grade 0 and I of OA versus grade II changes at 2 years postoperatively [32]. A latter study from the same group, reported successful clinical outcomes even in the presence of Tönnis grade II radiographic features [13]. Procedural success of arthroscopic management of FAI seems to be less dependent on the technical aspects of performing the procedure and more substantially dependent on patient selection and expectations [19].

The strengths of our study will be represented by rigorous methodology and selection criteria reported in line with international standards [16]. Appropriate quality assessment will allow objective evaluation of the evidence level. There will be a tenacious stratification based on stage of the disease even in the presence of a variety of terminology to report on joint degeneration and cartilage damage.

This systematic review will be the first step in a series of studies that will try to elucidate the most optimal treatment protocol and patient selection in the management of patients with FAI and concomitant moderate to advanced hip osteoarthritis in the young adult.
